# The Concentration of Large Extracellular Vesicles Differentiates Early Septic Shock From Infection

**DOI:** 10.3389/fmed.2021.724371

**Published:** 2021-09-16

**Authors:** Latthawan Monnamorn, Chutima Seree-aphinan, Piyatida Molika, Polathep Vichitkunakorn, Kovit Pattanapanyasat, Bodin Khwannimit, Raphatphorn Navakanitworakul

**Affiliations:** ^1^Faculty of Medicine, Department of Biomedical Sciences and Biomedical Engineering, Prince of Songkla University, Songkhla, Thailand; ^2^Faculty of Medicine, Department of Internal Medicine, Prince of Songkla University, Songkhla, Thailand; ^3^Faculty of Medicine, Department of Family and Preventive Medicine, Prince of Songkla University, Songkhla, Thailand; ^4^Faculty of Medicine Siriraj Hospital, Department of Research and Development, Mahidol University, Bangkok, Thailand

**Keywords:** septic shock, sepsis, extracellular vesicles, microvesicles, microparticles, imaging flow cytometry

## Abstract

Septic shock represents a subset of sepsis with severe physiological aberrations and a higher mortality rate than sepsis alone. Currently, the laboratory tools which can be used to identify the state of septic shock are limited. In pre-clinical studies, extracellular vesicles (EVs), especially large EVs (lEVs), have been demonstrated a role as functional inflammatory mediators of sepsis. However, its longitudinal trend during the disease course has not been explored. In this study, the quantities and subtypes of plasma-derived lEVs were longitudinally compared between patients with septic shock (*n* = 21) and non-sepsis infection (*n* = 9), who presented within 48 h of their symptom onset. Blood specimens were collected for seven consecutive days after hospital admission. lEVs quantification and subtyping were performed using an imaging flow cytometer. The experiments revealed a higher lEVs concentration in septic shock patients than infected patients at the onset of the disease. In septic shock patients, lEVs concentration decreased over time as opposed to infected patients whose lEVs concentration is relatively static throughout the study period. The major contributors of lEVs in both septic shock and infected patients were of non-leukocyte origins; platelets, erythrocytes, and endothelial cells released approximately 40, 25, and 15% of lEVs, respectively. Among lEVs of leukocyte origins, neutrophils produced the highest number of EVs. Nevertheless, the proportion of each subtype of lEVs among the given amount of lEVs produced was similar between septic shock and infected patients. These findings raise the possibility of employing lEVs enumeration as a septic shock identifying tool, although larger studies with a more diverse group of participants are warranted to extrapolate the findings to a general population.

## Introduction

WHO recognizes sepsis as a global health priority (1). Sepsis and septic shock are clinical syndromes defined as life-threatening organ dysfunction caused by dysregulated host immune response to infection; infection *per se* may not cause sepsis (2, 3). Septic shock represents a subset of sepsis with severe physiological aberrations resulting in increased mortality compared to sepsis alone (2). Systemic activation of various immune cells underlies the mechanisms in the pathogenesis of sepsis and septic shock; these cells produce an excessive amount of inflammatory mediators that induce endothelial injuries, coagulation abnormalities, and cellular damage, leading to end-organ dysfunction (4, 5).

Extracellular vesicles (EVs), heterogeneous membrane-bound vesicles released by various types of normal or diseased cells into the circulating system, is one of the mediators released by inflammatory cells in response to sepsis and septic shock (6). Large EVs (lEVs), originated from leukocytes, endothelial cells, and platelets, have been proposed as a means of intercellular communication that transfers inflammatory signals to orchestrate cytokine production, adhesion molecule expression, endothelial damage, and multiorgan dysfunction (6–9). However, some drawbacks limit the use of these interesting findings in clinical practice. Firstly, existing studies compared the characteristic of EVs between sepsis patients and healthy volunteers. The result gained from this design is a mixed effect of infection and sepsis rather than sepsis itself. As pointed out by the latest sepsis definition (2) and demonstrated by a published work of our group (10), immune alterations that occurred in sepsis and infection differ and should be addressed by the study design. In addition, the cross-sectional nature of previous studies omits the dynamicity of sepsis; hence, their results may not be able to apply to patients presenting at different time points during the disease course.

Therefore, in the present study, we aim to longitudinally compare the concentration and subtypes of plasma-derived lEVs between septic shock and non-sepsis infected patients using imaging flow cytometry.

## Materials and Methods

### Study Design and Participants

This longitudinal observational study was conducted in Songklanagarind hospital, Prince of Songkhla University's teaching hospital in Thailand. Patients diagnosed with acute infection and presented within 48 h of their symptom onset were screened; septic shock patients (*n* = 21) and infected patients without sepsis (*n* = 9) were recruited. According to Sepsis-3 definition (2), patients with evidence of organ dysfunctions (Sequential Organ Failure Assessment (SOFA) scores ≥2), a requirement of vasopressor support to maintain mean arterial pressure ≥65 mmHg, and a serum lactate level >2 mmol/L were categorized as septic shock. Patients with SOFA score and qSOFA <2 are categorized as infected patients. In infected patients, the qSOFA score was monitored regularly to ensure they remained in non-sepsis status during the study period. Sepsis patients without septic shock were not included as their clinical presentations are frequently overlapped with other acute conditions (such as heart failure precipitated by infection or acute pulmonary embolism), and their inclusion may lead to misclassification of the study subjects. Other exclusion criteria were age 18 years or less, prior diagnosis of sepsis 3 months before the recruitment, autoimmune conditions, immunosuppressive drug use including >2 weeks of corticosteroid treatment, pregnancy, active malignancy, and HIV infection. All patients received a standard of care as outline by the Surviving Sepsis Campaign Bundles (11). Written informed consent was acquired for all participants. The study was approved by the Human Research Ethics Committee of the Faculty of Medicine, Prince of Songkla University, Thailand (REC 62-366-4-2).

### Specimen Collection and Storage

For each patient, 1 ml of a whole blood sample was collected once daily between 6.00 and 7.00 a.m. for seven consecutive days. The day of admission was defined as Day 1. The samples were transferred to the lab for immediate processing in acid citrate dextrose solution-containing tubes to prevent an *in-vitro* generation of EVs from platelets. The samples were firstly centrifuged at 1,500 g for 5 min. Subsequently, the supernatant (i.e., the cell-free plasma) was centrifuged at 2,500 g for 15 min twice to make platelet-free plasma (PFP). The PFP samples were collected in polypropylene cryotubes and preserved at −80°C awaiting further analysis. The samples were stored for 13.68 ± 3.16 months before the analysis. Each sample was thawed once.

### Extracellular Particles Characterization From Platelet-Free Plasma using Nanoparticle Tracking Analysis

Before proceeding to flow cytometry analysis, we first performed nanoparticle tracking analysis (NTA) on PFP as an exploratory experiment characterizing extracellular particles constituted in the patients' plasma to overview their concentration and size distribution. The measurements were performed with NanoSight NS300 (Malvern, WR, UK) and analyzed with NanoSight NTA software version 3.2. By tracking Brownian motions of the particles within the plasma, this system analyses the size distribution and concentration of all types of nanoparticles, ranges from 0.01 to 1 μm in size (12). To prepare PFP samples for the analysis, thawed PFP samples were diluted in distilled water to the final volume of 1 ml. Two dilutions, ranged from ~1:8,000 to 1:250, were made. These dilutions achieved a particle concentration of 10^7^-10^9^ particles/ml (i.e., 20–100 particles/frame). The data were recorded with the camera set at level 14, the detection threshold was set at 4, and video capture of 5 cycles (30 s/cycle). The absolute concentrations of the particles in each dilution were calculated according to its dilution factor.

### Subtype Characterization of Extracellular Vesicles

#### Concentration and Separation of Extracellular Vesicles From PFP Samples

The differential centrifugation technique was employed for isolating lEVs from PFP samples. Firstly, thawed PFP samples were centrifuged at 2,000 g for 15 min. The supernatant was collected and re-centrifuged at 20,000 g for 45 min at 4°C. After the supernatant was removed, pellets were resuspended in 50 μL of phosphate-buffered saline (PBS). Western blot and electron microscopic study using JEM-2101 transmission electron microscope (TEM) were performed to confirm the presence of lEVs in the isolates. For electron microscopic examination, the isolates were fixed in 2.5% glutaraldehyde for 15 min and loaded on the carbon-coated copper grids. The grids were washed with Dulbecco's PBS first, followed by distilled water. The grids were then stained with 1% uranyl acetate for 10 min before the examination. For western blotting, the standard SDS-PAGE method was performed with cluster of differentiation CD9 (Cell Signaling, MA, US) and CD63 (Cell Signaling, MA, US) positivity as markers for identifying EVs, cytochrome C1 (Biolegend, CA, US) negativity to confirm the absence of co-isolated contaminated cell debris. ApoA1 (Abcam, CB, UK) were used to demonstrate lipoprotein contamination.

#### Quantification and Subtyping of Extracellular Vesicles by an Imaging Flow Cytometry

##### Performance Validation of the Imaging Flow Cytometry in Detecting lEVs

The experiments were done on Amnis® ImageStream®^X^ Mk II imaging flow cytometer (Luminex Corporation, TX, US) with 488 nm, 642 nm, and 785 nm lasers. The machine was calibrated regularly as per the manufacturer's instruction. The fluidics was set at low flow with high sensitivity and 40X magnification objective. The optimal antibody concentration, which gave positivity while minimizing image oversaturation, was titrated for all antibodies used in our experiments. We conducted a series of validation experiments prior to the study of EVs subtypes. The capability of the flow cytometer in detecting submicron particles was validated using a solution containing a mixture of polystyrene beads in 50, 100, 200, 400, and 700 nm sizes at a 2:2:2:1:1 ratio (Supplementary Figure 1). All antibody solutions were centrifuged at 17,000 g for 10 min before use to prevent antibody aggregation. Moreover, we confirmed the absence of false-positive events from contaminants and antibody clumps by examining the solutions with the flow cytometer (Supplementary Figures 2A–C). Additionally, we ensured that anti-human CD9-FITC antibodies (ImmunoTools, Friesoythe, Germany), a marker for EVs used in our study, only stained CD9 surface molecules by incubating the antibody with the samples containing blood cells isolated from whole blood of a healthy volunteer, EVs, and EVs permeabilized with Triton™ X-100 solution (Supplementary Figures 2D–F). CD9 positivity was detected from the samples containing blood cells and EVs but not the ones with permeabilized EVs; these experiments also functioned as a positive control. Background fluorescent intensities collected from unstained EVs samples were employed as a negative control.

##### Preparing Samples for Flow Cytometry

Our specimen preparation protocol was modified from the methods suggested by Headland et al. (13). The cellular origin of plasma derived EVs were identified by the following CD markers; CD235 for erythrocytes, CD41A for platelets, CD146 for endothelial cells, CD66b for neutrophils, CD14 for monocytes, CD19 for B lymphocytes, and CD3 for T lymphocytes. Five microliters of the isolated EVs samples were diluted with PBS to a total volume of 50 μL. The samples were incubated with an antibody cocktail, comprised of anti-human CD9-FITC (ImmunoTools, Friesoythe, Germany), anti-human CD235-PE (BD Pharmingen, NJ, US), anti-human CD41A-PE-Cy5 (BD Pharmingen, NJ, US), anti-human CD146-PE-Cy7 (BD Pharmingen, NJ, US), anti-human CD66b-PE (BD Pharmingen, NJ, US), anti-human CD14-PECF594 (BD Pharmingen, NJ, US), anti-human CD19-PERCP-Cy5.5 (BD Pharmingen, NJ, US), and anti-human CD3-APC-Cy7(BD Pharmingen, NJ, US), for 1 h in the dark at room temperature. Flow cytometry data acquisition was done immediately after the antibody incubation process.

##### Flow Cytometry Data Acquisition and Analysis

**Figure 1:**
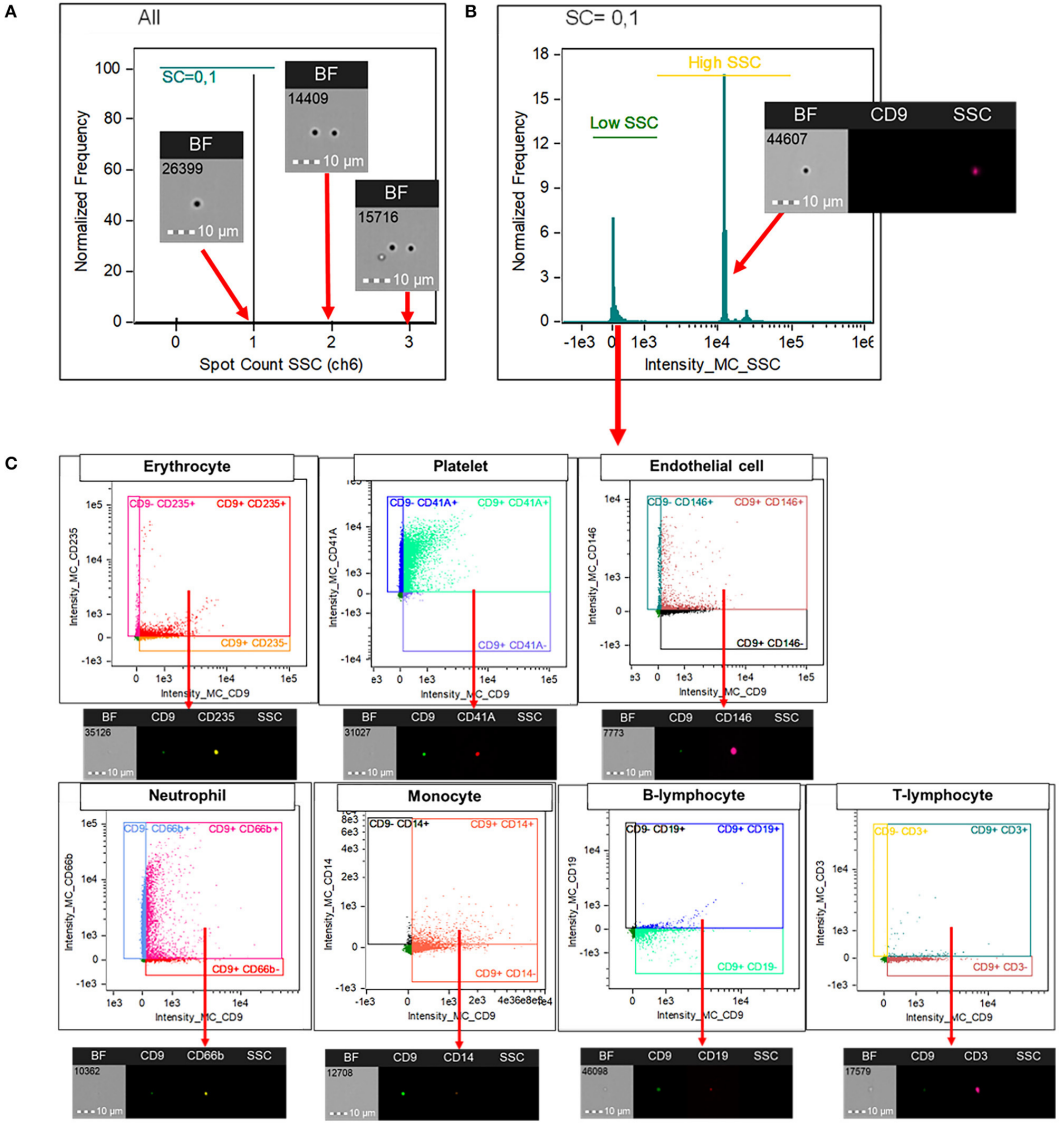
Imaging flow cytometry analysis for the identification of EVs' subtypes. Image Data Exploration and Analysis Software (IDEAS®) version 6.2 was used for the analysis. Doublets were excluded using “Spot count” on SSC channel **(A)**. This function is an image-based propriety software function that is suitable for counting events that appear as spots (e.g., parasite, phagocytosed particles) rather than a ring of fluorescent cell membrane. Events with spot counts of more than one was categorized as doublets and excluded from the analysis. Among singlets, large EVs were gated based on their intrinsically low SSC intensity **(B)**. Extracellular vesicles, subtyped by their cellular origin, were identified with a double positivity of CD9 and their corresponding markers; CD235 for erythrocytes, CD41A for platelets, CD146 for endothelial cells, CD66b for neutrophils, CD14 for monocytes, CD19 for B lymphocytes, and CD3 for T lymphocytes **(C)**. CD, cluster of differentiation; SSC, side scatter; EV, extracellular vesicles.

INSPIRE® ImageStream^X^ MKII software and Image Data Exploration and Analysis Software (IDEAS®) version 6.2 was used for flow cytometry data acquisition and analysis, respectively. The analysis software includes basic functions (as in traditional flow cytometer) and image-oriented proprietary functions. In the following description of the gating strategy, propriety software functions are italicized. The programmed was set to include speed beads during data acquisition; this machine use speed beads to calibrate its camera focus and the default setting will not count particles with their sizes comparable to speed beads (e.g., EVs) as events. We have collected at least 50,000 events from each experiment for further analysis. Events with adequate camera focus, as assessed by *Gradient RMS* > 50, were included for analysis. Firstly, the doublets were excluded using *Spot count* on side scatter (SSC) channel (Figure 1A). This function is an image-based function that is suggested to be suitable for counting events that appear as spots (e.g., parasite, phagocytosed particles) rather than a ring of fluorescent cell membrane (14). Events with spot counts of more than one was categorized as doublets and excluded from the analysis. Secondly, side scatter intensity was used to indicate EVs population as they have intrinsically low SSC intensity compared to speed beads with a high SSC intensity (Figure 1B). Finally, EVs, subtyped by their cellular origin, were identified with a double positivity of CD9 and their corresponding markers as described above (Figure 1C).

### Statistical Analysis

Statistical analyses were performed with STATA 16.1 (StataCorp LLC, TX, US) and GraphPad Prism 9.0.0 (GraphPad Software Inc. La Jolla, CA). Baseline clinical data including age, gender, comorbidities, source of infection, the presence or absence of bacteraemia, initial complete blood count, Acute Physiology and Chronic Health Evaluation (APACHE) II score and SOFA score on admission were collected. Categorical variables were compared using Fisher's exact test. Since all continuous data within our study were not normally distributed, they were presented as medians and interquartile ranges and compared between septic shock and infected patients using Wilcoxon rank-sum tests. All tests with a *p*-value below 0.05 were considered statistically significant.

## Results

### Characteristics of Study Participants

We followed the clinical status of the study participants for seven consecutive days. Compared to non-sepsis patients, the study participants with septic shock had a substantially higher APACHEII and SOFA score on admission (Table 1). Among septic shock patients, three died before Day 7, one was discharged on Day 6 due to early recovery, and the rest remained ill on Day 7. None of the infected patients developed clinical signs or symptoms of sepsis; their qSOFA scores were <2 throughout the study period. Early discharge and deaths of the study participants resulted in 14 (6.67%) missing observations. Baseline characteristics, comorbidity profile, source of infection, and the full blood count's composition of septic shock and infected patients were similar. Most patients suffered either respiratory tract infection, gastrointestinal infection, or urinary tract infection. There was a trend toward a higher rate of bacteraemia among septic shock patients than infected patients; however, this observation was not statistically significant.

**Table 1:** Basic characteristics of study subjects ^†^ (*n* = 30).

**Characteristics**	**Infection** **(** ***n*** **= 9)**	**Septic shock** **(** ***n*** **= 21)**	***p*** **-value**
Age, median (IQR)	78 (72–80)	78 (70–83)	1.000^a^
Sex, male (%)	56	52	1.000^b^
Bacteremia (%)	22	47	0.249^b^
**Source of infection (%)**			
Respiratory tract	45	38	1.000^b^
Gastrointestinal	22	29	1.000^b^
Urinary tract	22	19	1.000^b^
Others	11	14	1.000^b^
**Co-morbidities (%)**			
Cardiovascular diseases	56	62	1.000^b^
Diabetes mellitus	33	43	0.704^b^
Respiratory diseases	22	29	1.000^b^
Neurological diseases	44	24	0.389^b^
Renal diseases	33	10	0.143^b^
Liver diseases	0	19	0.287^b^
Hematological diseases	0	10	1.000^b^
**Full blood count on admission, median (IQR)**			
White blood cells (103/μL)	12.71 (10.41–18.31)	10.21 (5.99–17.87)	0.483^a^
Red blood cell (106/μL)	4.38 (4.20–5.23)	4.01 (3.16–4.47)	0.153^a^
Platelets (103/μL)	172 (148–238)	149 (97–210)	0.441^a^
Neutrophils (%)	82.3 (75–84.4)	72 (52–88.1)	0.428^a^
Lymphocytes (%)	9.9 (8.1–11.9)	7.5 (5–14)	0.634^a^
Monocytes (%)	4.8 (2.1–5.7)	3.5 (1–6)	0.429^a^
Admission APACHE II score, median (IQR)	11 (9–20)	29 (23–34)	0.000^*^^a^
Admission SOFA score, median (IQR)	1 (1–1)	11 (10–13)	0.000^*^^a^

† *All study subjects had acute infection and presented to the hospital within 48 h of the symptom onset. APACHE, Acute Physiology and Chronic Health Evaluation; IQR, interquartile range; SOFA, Sequential Organ Failure Assessment*.

a *Wilcoxon rank-sum test*.

b *Fisher's exact test*.

* *p < 0.05*.

### The Quantities and Sizes of Extracellular Particles From Septic Shock and Infected Patients

**Figure 2:**
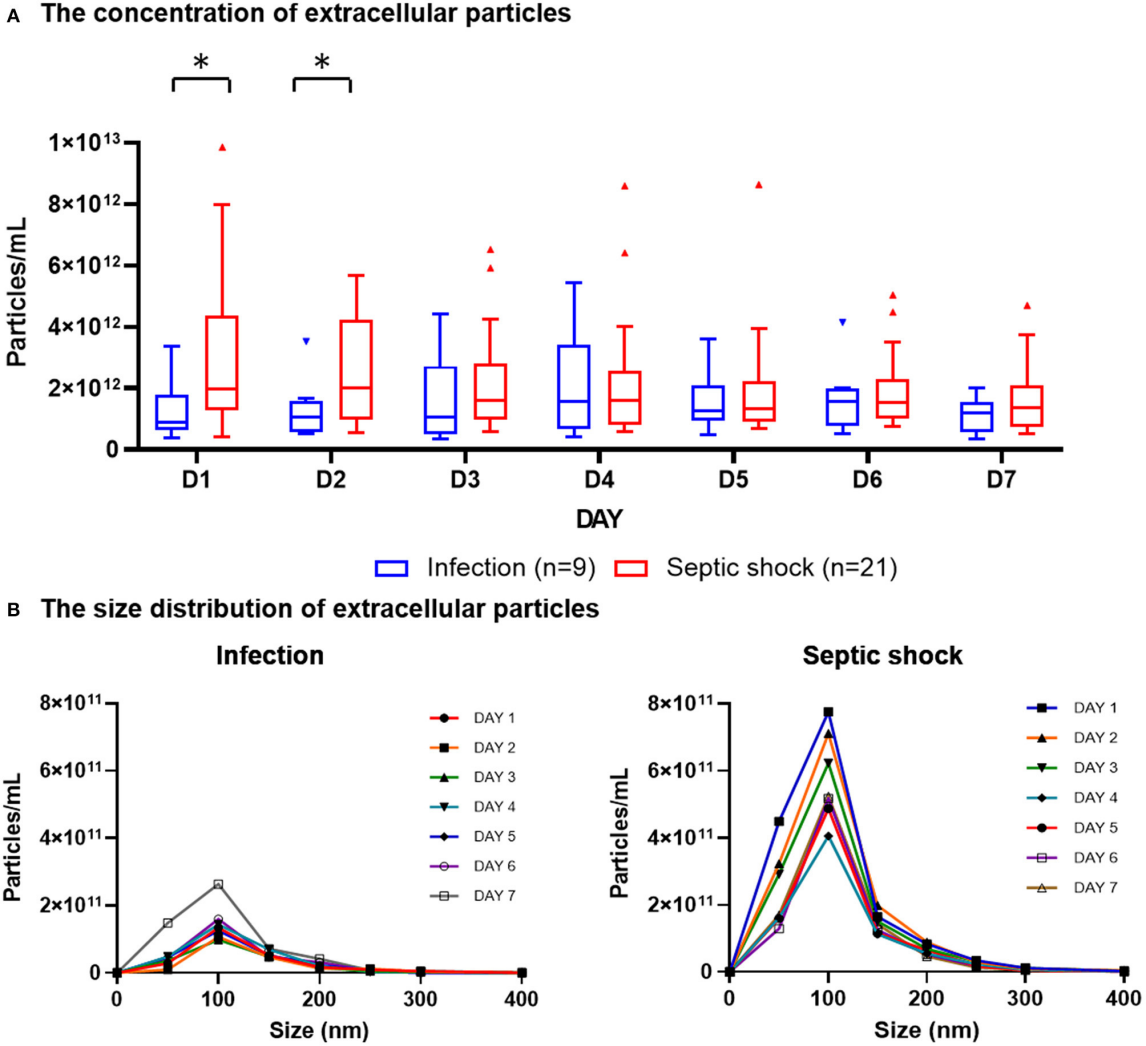
Plasma concentration and size distribution of extracellular particles in PFP samples of patients with septic shock and infection. PFP samples were collected daily for seven consecutive days after hospital admission for analysis. Nanoparticle tracking analysis was employed to measure the concentration of extracellular particles of varying sizes within PFP samples. The daily concentrations of extracellular particles were presented in boxplot graphs using median and interquartile range **(A)**. The particles varied in sizes, with the majority of them had a diameter of approximately 100 nm **(B)**. Wilcoxon rank-sum tests were performed to compare the concentration of extracellular particles between septic shock and infected patients. *p-value < 0.05 for Wilcoxon rank-sum tests. PFP, platelet-free plasma.

The quantities and sizes of extracellular particles were measured from platelet-free plasma in order to recover the highest amount of extracellular materials for the analysis. We found that, on admission, patients with septic shock demonstrated a higher plasma concentration of extracellular particles compared to infected patients without sepsis (Figure 2A). The discrepancy between septic shock and infected patients were most pronounce and statistically significant during the first 2 days of the disease's course. From Day 3 onwards, the quantity of extracellular material virtually equalized between these two groups of patients. The extracellular particles within the patient's plasma varied in size, with the majority of them had a mean diameter of 100 nm (Figure 2B). Among all extracellular particles detected in the patients' plasma, the proportion of extracellular particles with the size of 1–100 nm, 101–200 nm, and >200 nm were approximately 70, 30, and 3%, respectively (Table 2). These proportions of extracellular particles of specific sizes were similar across the days during the study periods.

**Table 2:** Concentrations and sizes of extracellular particles in PFP samples of patients with septic shock and infection^†^.

**Day after diagnosis**	**Particles concentration (objects × 10^11^/mL) Median (IQR)**		**The proportion of the particles according to their sizes (%)**					
			**1–100 nm**		**101–200 nm**		**>200 nm**	
	**Infection**	**Septic shock**	**Infection**	**Septic shock**	**Infection**	**Septic shock**	**Infection**	**Septic shock**
1	9.00 (6.62**–**14.80)	^*^19.82(14.20**–**41.64)	65.62	80.45	27.89	16.26	6.50	3.29
2	10.60 (6.12**–**15.10)	^*^20.30 (10.80**–**42.00)	60.07	75.69	30.82	21.05	9.11	3.26
3	10.60 (5.63**–**22.90)	16.10 (9.82**–**26.30)	65.11	77.48	30.91	18.63	3.98	3.90
4	15.50 (7.12**–**19.70)	16.80 (8.56**–**23.90)	66.40	74.08	31.23	21.78	2.37	4.15
5	12.50 (11.70**–**16.80)	12.80 (9.20**–**20.50)	67.97	75.94	29.46	21.11	2.57	2.95
6	15.60 (8.00**–**19.70)	15.70 (10.40**–**21.60)	70.38	76.01	27.36	20.94	2.27	3.05
7	11.80 (6.58**–**14.00)	13.80 (8.32**–**18.90)	76.18	76.85	20.98	20.93	2.84	2.21

† *PFP samples were collected daily for seven consecutive days after hospital admission for analysis. Nanoparticle tracking analysis was employed to measure the concentration of extracellular particles of varying sizes within PFP samples. Wilcoxon rank-sum tests were performed to compare the concentration of extracellular particles between septic shock and infected patients*.

* *p-value < 0.05 for Wilcoxon rank-sum tests*.

*IQR, interquartile range; PFP, platelet-free plasma*.

### Quantification and Subtyping of lEVs From Septic Shock and Infected Patients

**Figure 3:**
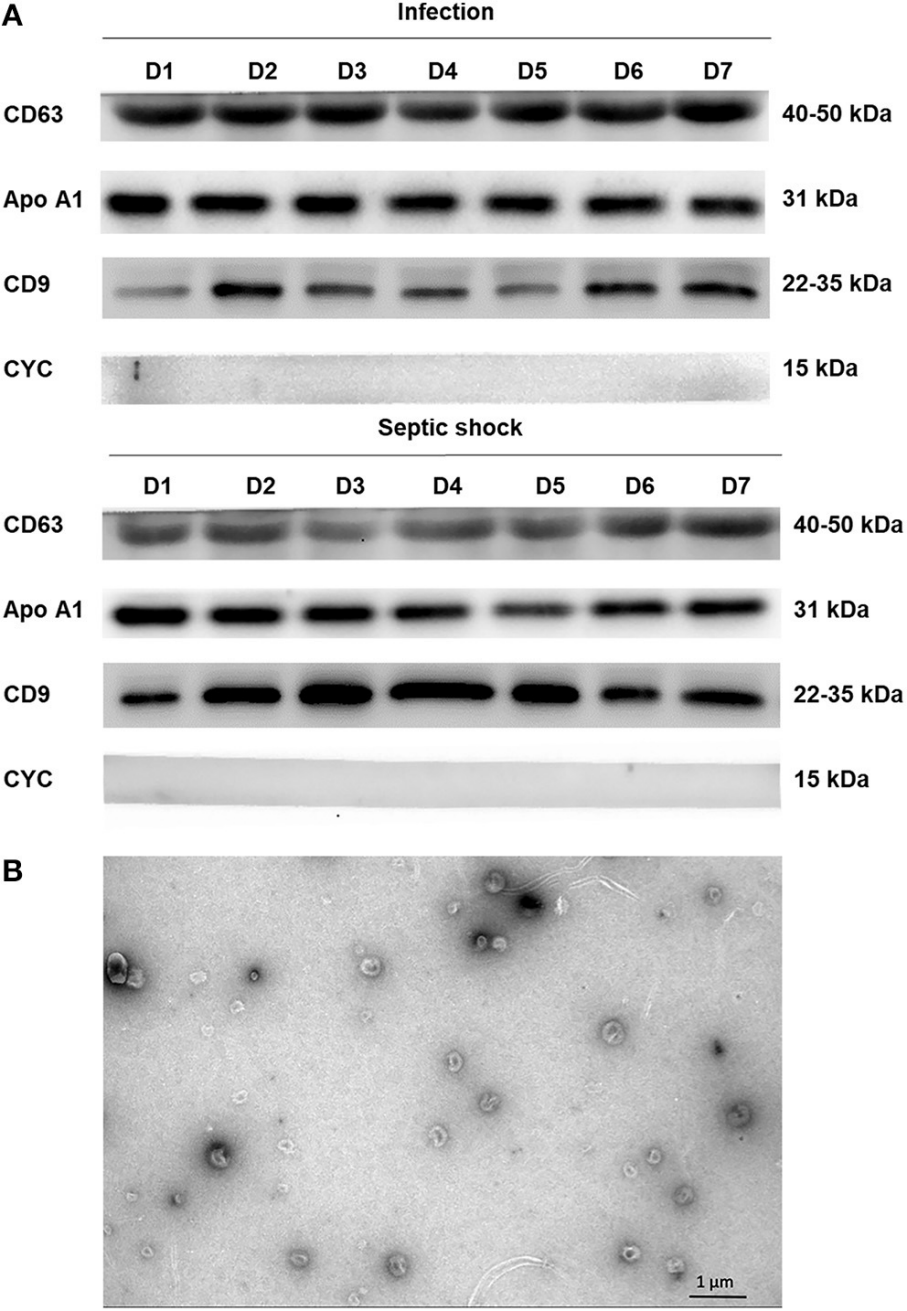
Assessing extracellular vesicle isolates by western blot and electron microscopy. Using the differential centrifugation technique, EVs were concentrated and separated from PFP samples of septic shock and infected patients. The isolates were examined with western blot using the standard SDS-PAGE method and JEM-2101 transmission electron microscope (TEM). Western blot confirmed the presence of extracellular vesicles (i.e., CD9, CD63 positivity) and the absence of co-isolated contaminants of cell debris (i.e., CYC1 negativity) **(A)**. As demonstrated by ApoA1 positivity, there was some degree of lipoprotein contamination within the isolates from both septic shock and infected patients. TEM illustrated numerous single EVs and the absence of protein aggregates or other amorphous substances within the isolates **(B)**. Morphologically, EVs appeared as a cup-shaped structure with an approximate size of around 100–200 nm. ApoA1, Apolipoprotein A1; CD, cluster of differentiation; CYC1, cytochrome C1; EV, extracellular vesicle; PFP, platelet-free plasma.

**Figure 4:**
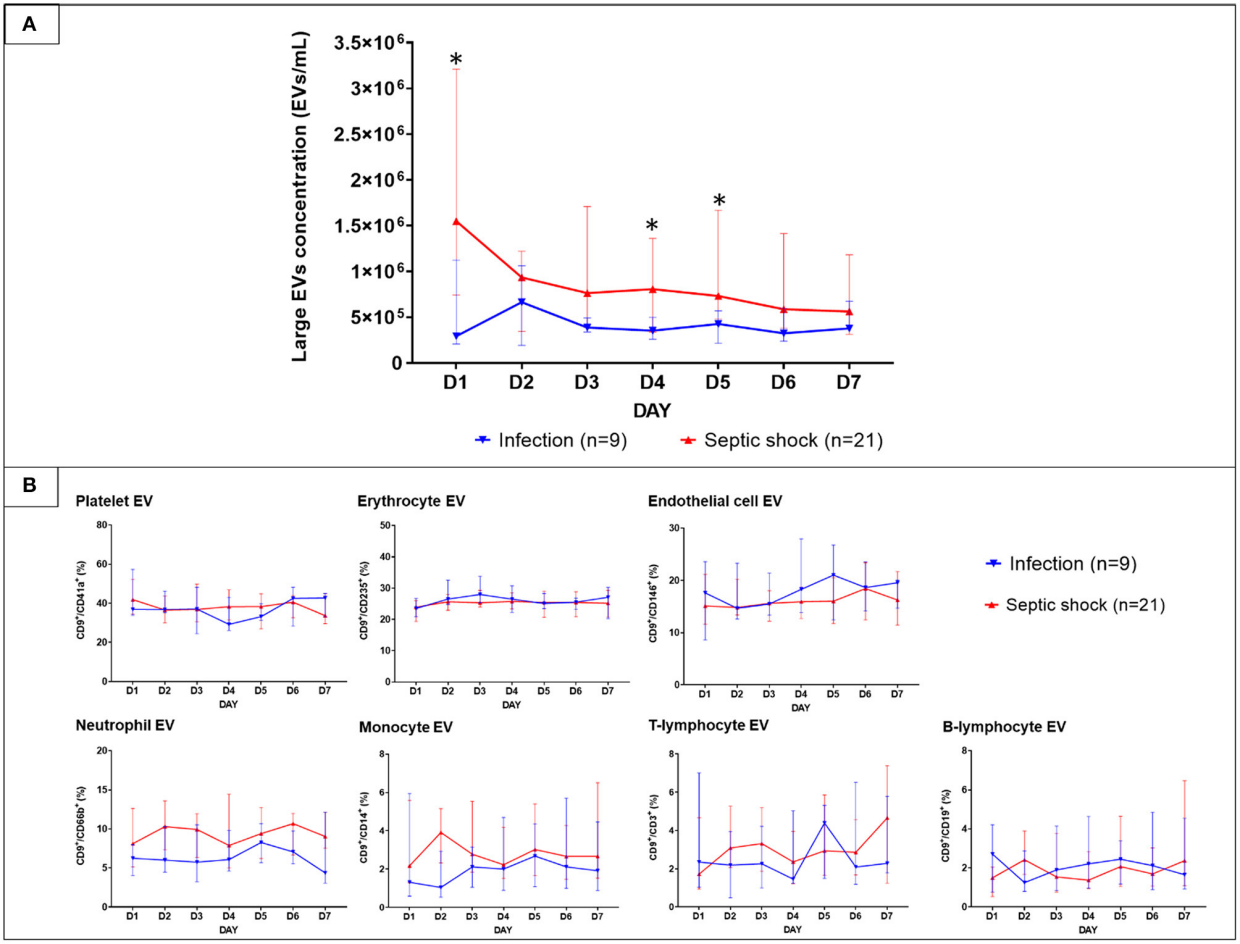
The longitudinal trends of large EVs concentration and the proportion of each subtype in patients with septic shock and non-sepsis infection. EVs were isolated from PFP samples collected daily for seven consecutive days after hospital admission. Large EVs concentration **(A)** and their proportion **(B)** sorted by subtypes were quantified by the imaging flow cytometry analysis. The results were illustrated in line graphs with error bars using median and IQR. The comparison of the results between septic shock and non-sepsis patients were performed with Wilcoxon rank sum tests. *p-value from Wilcoxon rank-sum test <0.05. EVs, extracellular vesicles; IQR, interquartile range; PFP, platelet-free plasma.

With the use of differential centrifugation, we aimed to recover samples with a high concentration of lEVs with the least possible non-vesicular components. The isolates from septic shock and infected patients were examined with western blot and TEM for purity assessment (Figure 3). Western blot confirmed the presence of EVs (i.e., CD9, CD63 positivity) and the absence of co-isolated contaminants of cell debris (i.e., CYC1 negativity). Additionally, TEM illustrated numerous single EVs and the absence of protein aggregates or other amorphous substances within the isolates (Figure 3B). Although present in the samples, lipoproteins did not interfere with flow cytometry analysis under our flow cytometry gating strategy described. The isolates underwent flow cytometric quantification and characterization of lEVs by protein markers that represent their cell origins. Similar to the trend we found on extracellular particles, on admission, a larger amount of lEVs were retrieved from the plasma of patients with septic shock compared to infected patients without sepsis (Figure 4A). In septic shock patients, the amount of lEVs was highest on Day 1 and gradually declined during the study period. On the contrary, the number of lEVs was considerably lower for infected patients than septic shock patients, with some degree of fluctuation throughout the study period. The statistically significant difference in the number of lEVs between septic shock and infected patients can be demonstrated in Day 1, Day 4, and Day 5. Interestingly, regarding the EV subtypes, the major contributors of lEVs in both septic shock and infected patients were of non-leukocyte origins (Figure 4B). During the study period, platelets, erythrocytes, and endothelial cells released ~40, 25, and 15% of lEVs, respectively. Among lEVs of leukocyte origins, neutrophils produced the highest number of EVs. However, no statistical significance could be demonstrated from the daily comparisons of the proportion of each subtype of lEVs among the given amount of lEVs between septic shock and infected patients.

## Discussion

Numerous publications suggested the use of EV monitoring in the identification of sepsis patients (6). However, implementing these studies into the current clinical practice could be problematic because they performed the tests in the participants defined by the old sepsis definition and omitted the chronological progression of the disease by studying EVs at only specific time points (6). Also, as sepsis and infection are now recognized as different entities (2), the distinction of EV character between sepsis and infection cannot be demonstrated by these previous studies, which compared sepsis patients to controls without infections (e.g., healthy volunteers, critically-ill patients). To fill these knowledge gaps, we designed a longitudinal study to compare the quantity and subtypes of extracellular vesicles between “infected” patients with or without sepsis. Our case definition aligned with the newest sepsis definition (Sepsis-3). We also synchronized the time course of the disease between septic shock and non-sepsis patients; all study subjects entered the study within 48 h of their symptom onset. This design seeks to evaluate whether the process of septic shock (in addition to the effect of infection) produces a specific pattern of changes in EVs' character.

Our study demonstrated that the most striking feature that distinguishes septic shock from non-sepsis patients is the number of lEVs at the onset of the disease. We found that the number of EVs released in response to septic shock is higher than those released in response to infection without sepsis. The difference in the number of lEVs between septic shock and infected patients was most prominent at the onset of the disease and gradually reduced as the patients progress through their clinical course. The observed longitudinal changes of plasma lEVs concentration is similar to many biologically active compounds with elevated levels during sepsis (e.g., c-reactive protein, interleukin 6) (15–17). Given that point-of-care EV quantification tests are under development (18), our findings suggest that lEVs quantification could be one of the means to identify patients with septic shock early in its course. The specificity of EV to sepsis may raise concerns regarding the use of EVs to diagnose septic shock since EV concentration may rise in various conditions (19), we believe that a disease marker does not need to be specific to be helpful. Several clinical studies which performed EV enumeration using various techniques demonstrated that the number of EVs could be used to differentiate sepsis patients from healthy controls (20, 21), critically-ill patients without infection (22), and patients with non-infectious systemic inflammatory responses (23). Our study adds infection without sepsis into the list of conditions in which the plasma concentration of EV may help distinguish them from septic shock. Other sepsis markers we used in clinics nowadays (such as c-reactive protein, procalcitonin, serum lactate) are far from being specific to sepsis, but they have been useful in diagnosing sepsis patients (24). Serum lactate, in particular, is included in the diagnostic algorithm of septic shock despite their elevation in other conditions such as cardiogenic shock and hypoxemia (25). Hence, EV quantification could be a good addition to the list of tests that aid the diagnosis of septic shock, especially in a situation when the interpretation of other tests is problematic (e.g., serum lactate in patients with mixed causes of shock, c-reactive protein in patients with autoimmune diseases).

A quest to seek the signature biomarkers of sepsis is still ongoing; EV characterization, either by their cell origins, specific surface markers, or their miRNA contents, is an area that has been receiving interest in this matter. Numerous EV subtypes were linked to various aspects of sepsis pathobiology (26); some of them promotes the inflammatory reactions of sepsis (27–32) while others alleviate the damage from sepsis (33–38). Our study found that, in septic shock patients, most plasma-derived EVs are of non-leukocyte origins (i.e., platelet-EVs, erythrocyte-EVs, endothelial cells-EVs; ranked in descending order of plasma concentration). Interestingly, neutrophil-derived EVs only constitute a minority of plasma EVs despite the existing sepsis and infection-induced neutrophil dysfunctions (10) and previous publications highlighting the association between neutrophil EVs and the inflammatory process of sepsis (26, 27). These findings emphasize an important role of blood cells of non-leukocyte origin in modulating and propagating the hyperinflammatory reactions of sepsis and infection. Platelet-derived EVs has been proposed as a cause of sepsis-induced coagulation abnormalities (32), and endothelial cell-derived EVs were shown to exert protective effects against sepsis-related organ dysfunctions (33–35). Even though the position of erythrocyte-derive EVs in sepsis inflammatory cascades is poorly described, the biophysical properties of erythrocytes are altered in the presence of sepsis-triggered plasma-derived extracellular vesicles (31); which may lower their threshold of EV release. Nonetheless, our study also demonstrated that, as far as the characterization of lEVs' cell origin is concerned, no EV subtype is sepsis-specific because both septic shock and infected patients had a similar proportion of each subtype of lEVs among the given amount of EVs being produced. In fact, in healthy adults, platelets and erythrocytes also produce the largest amount of EVs into plasma (39). Therefore, to differentiate sepsis from infection with EV characterization, a more in-depth analysis of EV subpopulation of the same cell origins may be needed to reveal some subtypes of EVs that are sepsis-specific. For instance, a study on neutrophil-derived EVs by Youn et al. (27) found that one subpopulation of neutrophil EVs [i.e., neutrophil-derived trails (NDTRs)] contains proinflammatory miRNA in contrast to the other [neutrophil-derived microvesicles (NDMVs)] that contain anti-inflammatory miRNA. Additionally, among sepsis-related endothelial cells derived EVs, selective miRNA-375-3p upregulation activates signaling pathways that relieve sepsis-related myocardial injury (33) while miRNA-93-5p upregulation confers protective effects to sepsis-induced acute kidney injuries (35). The main caveat for the clinical applications of these sepsis-related EV subtypes is that their scope of use is usually restricted to specific settings or scenarios (40, 41), hence limiting their generalisability to a heterogeneous group of sepsis patients. Also, compared to EV quantification, EV subtype characterization required more sophisticated, time-consuming laboratory methods, which may not be pragmatic for using in a fast-paced ICU. If one were to apply EV studies in clinical practice, lEVs quantification is a good option for EV studies to look into, until we can find a EV subpopulation that is truly sepsis-specific.

Our study has some limitations. Firstly, as we aimed to recover as many extracellular particles as possible for NTA analysis, the protein and lipid elimination processes were skipped. Although this is non-confirmatory by the methods, their proportion may be implied from NTA analysis results as minimal since the proportion of the particle of their size is low. Secondly, as previous publications have mainly validated flow cytometric EVs subtyping analysis for lEVs (42), our work preferentially studied lEVs. Hence, our observations are only applied to this EVs subpopulation and not to EVs of their sizes. Lastly, since patient heterogeneity has been a big obstacle for interpreting sepsis biomarker studies (43), we chose to conduct our experiments in a clinically homogenized, albeit small, group of patients. To achieve this, we excluded ones with autoimmune diseases, cancer, and HIV infection. Moreover, despite being a part of the disease continuum, sepsis patients were omitted from the study as their clinical presentation frequently overlapped or coincided with other acute conditions. Their inclusion may lead to misclassification of the study subjects and increased heterogeneity among patients in the same groups. Further studies in a larger and more diverse group of participants are warranted to extrapolate our findings to a general population and set baseline plasma lEVs concentration in infected patients with these excluded conditions.

## Conclusion

Septic shock patients had a higher lEVs concentration than infected patients without sepsis; the difference in the number of lEVs between septic shock and infected patients is most prominent at the onset of the disease and gradually reduces as the patients progress through their clinical course. Furthermore, the proportion of each subtype of lEVs among the given amount of lEVs being produced was similar between septic shock and infected patients. Our findings raise the possibility of employing lEVs enumeration, rather than lEVs subtyping, as a tool for aiding physicians in identifying patients with septic shock, although further studies in a larger and more diverse group of participants are warranted to extrapolate our findings to a general population.

## Data Availability Statement

The raw data supporting the conclusions of this article will be made available by the authors, without undue reservation.

## Ethics Statement

The studies involving human participants were reviewed and approved by the Human Research Ethics Committee of the Faculty of Medicine, Prince of Songkla University, Thailand (REC 62-366-4-2). The patients/participants provided their written informed consent to participate in this study.

## Author Contributions

LM, CS-a, and BK carried out sample collection and data acquisition. LM and PM performed the experiments. KP contributed reagents. LM, CS-a, and PV were responsible for data curation and formal analysis of the study. LM and CS-a, wrote the original draft of the manuscript. KP, PV, BK, and RN reviewed and edited the manuscript. RN supervised the project and funding acquisition. All authors contributed to the article, approved the submitted version, and took part in the conceptualization and methodology planning of the study.

## Funding

This work was financially supported by a research grant from the Faculty of Medicine, Prince of Songkla University (Grant No.62-366-4-2). LM is a recipient of a scholarship from the Faculty of Medicine, Prince of Songkla University.

## Conflict of Interest

The authors declare that the research was conducted in the absence of any commercial or financial relationships that could be construed as a potential conflict of interest.

## Publisher's Note

All claims expressed in this article are solely those of the authors and do not necessarily represent those of their affiliated organizations, or those of the publisher, the editors and the reviewers. Any product that may be evaluated in this article, or claim that may be made by its manufacturer, is not guaranteed or endorsed by the publisher.
