# Supplementary material for: The Concentration of Large Extracellular Vesicles Differentiates Early Septic Shock From Infection
Source: Front Med (Lausanne). 2021 Sep 16;8:724371. doi: 10.3389/fmed.2021.724371 (PMC8481381; doi:10.3389/fmed.2021.724371)
Supplement: Supplementary file 1 [file Data_Sheet_1.docx]

Supplementary Material

##
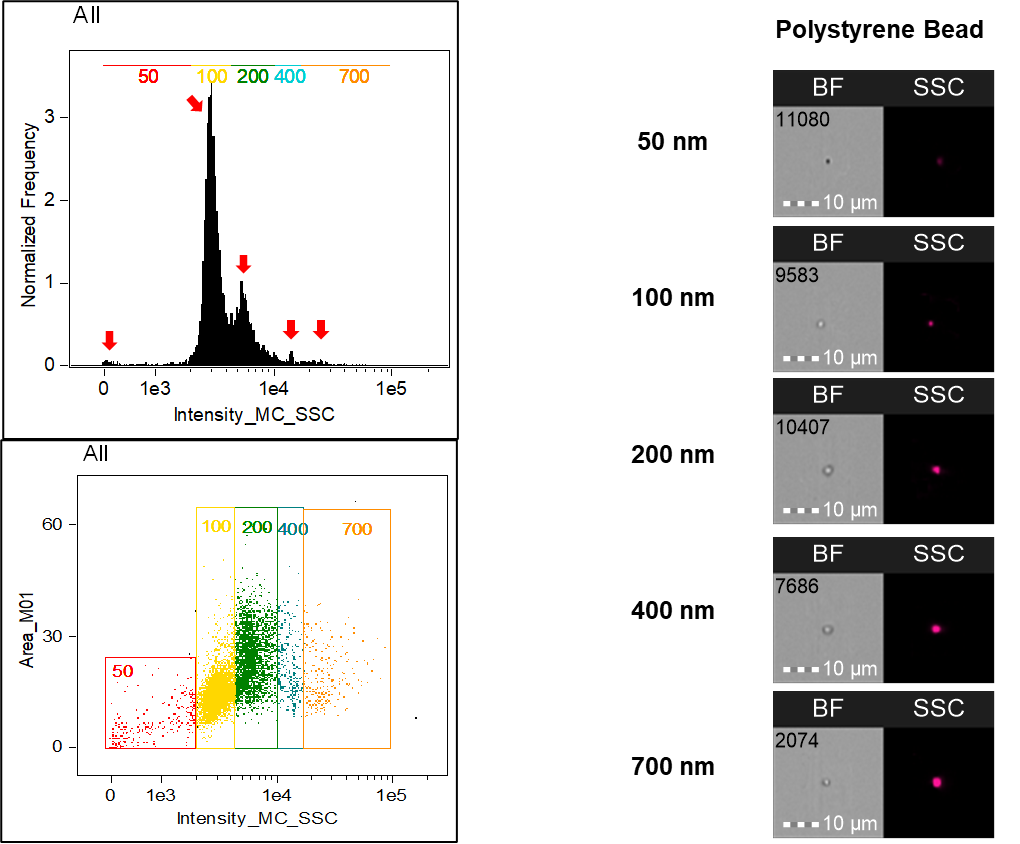
Supplementary Figures

**Supplementary Figures 1. Performance analysis of Amnis^®^ ImageStream^®X^ Mk II imaging flow cytometer: detection of submicron particles.** This analysis was performed on IDEAS^®^ software version 6.2; propriety software functions are italicized. 488 nm, 642 nm, and 785 nm lasers were employed for the experiments. The fluidics was set at low flow with high sensitivity and 40X magnification objective. The capability of the flow cytometer in detecting submicron particles was validated using a solution containing a mixture of 50, 100, 200, 400, and 700 nm polystyrene beads. Brightfield *Area*, which represents the particle sizes, and side scatter intensity were used to discriminate beads of varying sizes.



**
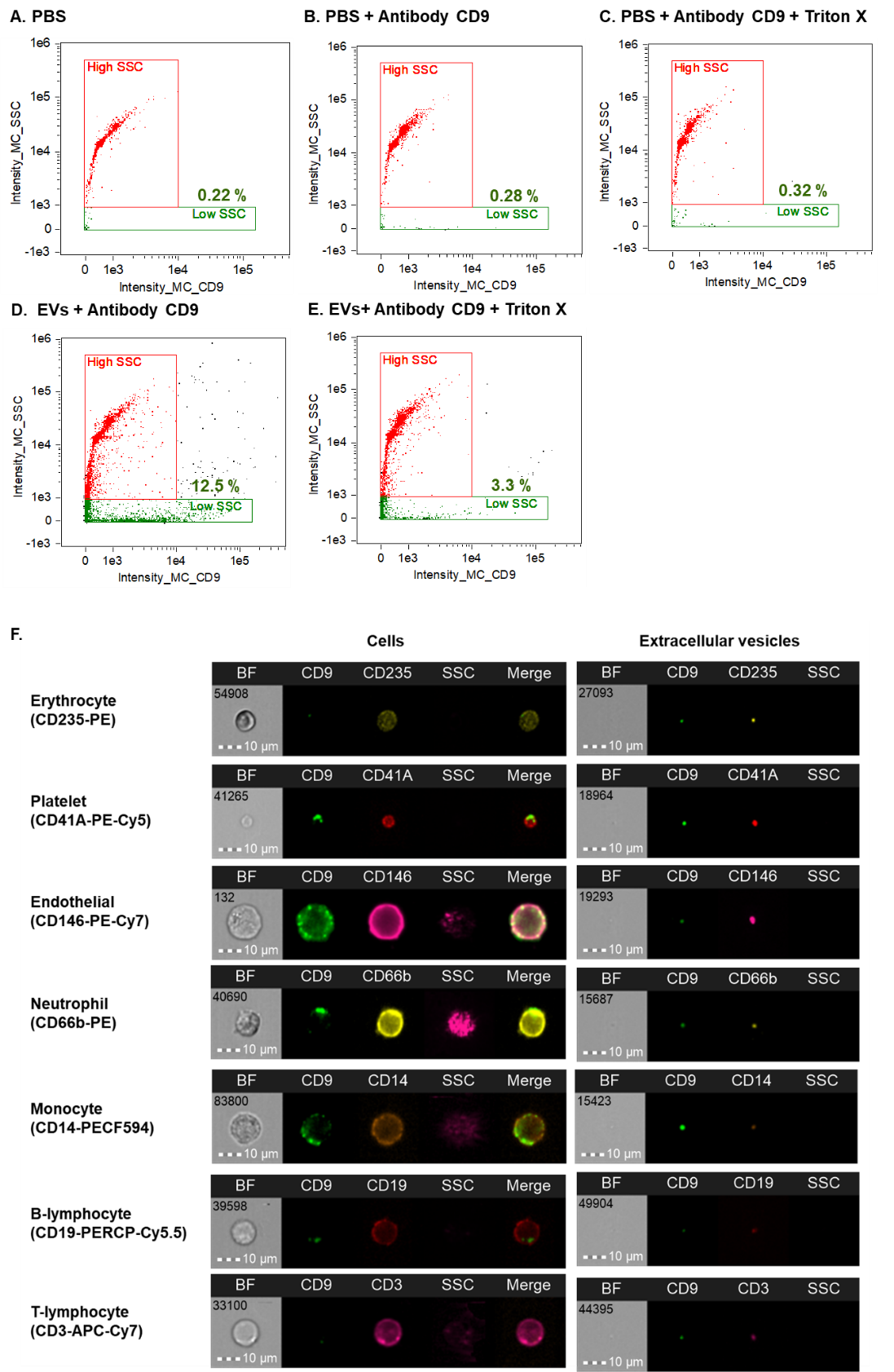
**

**Supplementary** **Figures 2. Performance analysis of Amnis^®^ ImageStream^®X^ Mk II imaging flow cytometer: detection of large EVs.** This analysis was performed on IDEAS^®^ software version 6.2. Speed beads and EVs can be differentiated by having high and low SSC intensity, respectively. Anti-human CD9-FITC antibody was used as a marker of EVs. The absence of false-positive events from contaminants and antibody clumps was confirmed by examining PBS, Triton-X, and anti-human CD9 antibody solution with the flow cytometer (A, B, C). The antibody solution was incubated with non-permeabilized and permeabilized EVs samples to ensure anti-human CD9 antibodies only stained CD9 surface molecules (D, E). CD9 positivity markedly reduced in the permeabilized EVs sample compared to the non-permeabilized one. For positive control experiments, the optimal antibody concentration was titrated on both blood cells and EVs; CD9, CD235, CD41, CD146, CD66b, CD14, CD19, CD3 were used as markers for EVs, erythrocytes, platelets, endothelial cells, neutrophils, monocytes, B lymphocytes, and T lymphocytes, respectively (F). Background fluorescent intensities collected from unstained EVs samples were employed as a negative control. Abbreviations: CD: cluster of differentiation, EVs: extracellular vesicles, PBS: phosphate-buffered saline, SSC: side scatter.
